# Characterizing Esophageal Motility in Neonatal Intensive Care Unit Patients Using High Resolution Manometry

**DOI:** 10.3389/fped.2022.806072

**Published:** 2022-02-14

**Authors:** Maissa Rayyan, Taher Omari, Veerle Cossey, Karel Allegaert, Nathalie Rommel

**Affiliations:** ^1^Neonatal Intensive Care Unit, University Hospitals Leuven, Leuven, Belgium; ^2^Department of Development and Regeneration, Katholieke Universiteit Leuven, Leuven, Belgium; ^3^College of Medicine and Public Health, Flinders University, Adelaide, SA, Australia; ^4^Clinical Pharmacology and Pharmacotherapy, Department of Pharmaceutical and Pharmacological Sciences, KU Leuven, Leuven, Belgium; ^5^Department of Hospital Pharmacy, Erasmus Medical Center University Medical Center, Rotterdam, Netherlands; ^6^Department of Neurosciences, Experimental Otorhinolaryngeal, Deglutology, KU Leuven, Leuven, Belgium; ^7^Neurogastroenterology and Motility, Gastroenterology, University Hospitals Leuven, Leuven, Belgium

**Keywords:** esophageal motility, dysphagia, infant, preterm, esophageal atresia, congenital diaphragmatic hernia, bronchopulmonary dysplasia

## Abstract

**Objective:**

To characterize esophageal motility and esophago-gastric junction (EGJ) function during feeding in neonatal intensive care unit (NICU) patients.

**Patients and Methods:**

High resolution manometry with impedance (HRIM) was used to investigate esophageal motility and EGJ function in patients admitted to the NICU. Twenty-eight preterm born infants with bronchopulmonary dysplasia (BPD), 12 born with isolated congenital diaphragmatic hernia (iCDH), and 10 with esophageal atresia (EA) were included. Thirteen healthy infants were included as controls. Esophageal motility and EGJ function were analyzed using objective esophageal bolus transport parameters.

**Results:**

Normal esophageal peristaltic wave patterns were observed in all investigated infants without EA. Nine of 10 patients with EA presented with abnormal esophageal motor wave patterns. A total of 224 nutritive swallows were analyzed (controls, *n* = 48; BPD, *n* = 96; iCDH, *n* = 60; EA, *n* = 20). Infants with BPD and iCDH had similar distal contractile strength (DCI) compared to healthy controls, while in patients with EA, DCI was significantly lower (Kruskal-Wallis test, *p* = 0.001). In most infants, EGJ relaxation after swallowing was unaffected. EGJ barrier function, in terms of EGJ-contractile integral, also appeared well-developed and did not differ significantly among patient groups.

**Conclusions:**

We conclude that esophageal motility studies using pressure-impedance analysis are feasible in young infants. Bolus transport mechanisms following nutritive swallows appeared well-established in all investigated infants with the exception of those with EA. EGJ relaxation was also functional after deglutition and EGJ function as an anti-reflux barrier appeared well-developed in all investigated NICU groups.

## Introduction

Infants admitted to the neonatal intensive care unit (NICU) commonly present with feeding problems, such as decreased oral intake and feeding intolerance and may suffer from complex diseases, involving prematurity or congenital malformations. The origin of such feeding problems may be disease-specific or can be secondary to numerous factors, including illness severity, associated medical conditions, or surgical interventions.

Preterm infants have an immature nervous system, leading to immature oral intake, and failure to coordinate sucking, swallowing, and breathing. Developmental changes in swallow physiology in the preterm infant have been described in infants under 34 weeks (1). In the youngest infants, low pharyngeal pressures are observed, as well as poor coordination with relaxation of the upper esophageal sphincter (UES). The physiology of distal esophageal motility in dry swallows was described in healthy, preterm infants two decades ago (2, 3). Additionally, differential development of the three contraction segments of the esophagus have been documented in preterm and term infants, using high resolution manometry (HRM) (4). Contraction in the second segment of the esophagus (proximal portion of smooth muscle esophagus) is present in almost all (pre) term infants, while the first (striated muscle) segment and distal portion of esophagus smooth muscle develop later. However, there are only few data relating to objective esophageal bolus transport parameters during nutritive swallows in preterm infants (5) and in preterm infants with feeding problems suffering from bronchopulmonary dysplasia (BPD) (6).

Additionally, data on esophageal function in infants born with congenital anomalies is scarce. In children with esophageal atresia (EA), motility disorders are likely primarily due to the disturbed development of the intrinsic innervation of the esophagus (7). End-to-end anastomosis of the esophagus can further disturb vagal innervations, vascular supply, or cause traction on the lower esophagus (8). Although respiratory and feeding problems, as well as gastro-esophageal reflux disease, are common in infants with isolated congenital diaphragmatic hernia (iCDH), little is known about the pathophysiology of their feeding problems (9, 10).

Over recent years, techniques to evaluate esophageal function have evolved, with solid state catheters becoming available in small sizes, which are more tolerable for patients, enabling the evaluation of the esophago-gastric junction (EGJ) in small infants. For data acquisition, HRM can be combined with impedance (HRIM). Subsequently, assessment of esophageal motility and bolus flow can be performed simultaneously using pressure-impedance analysis (11, 12).

The aim of this prospective study was to gain more knowledge on esophageal function during nutritive swallows in infants with associated medical pathology. Therefore, HRIM data until currently collected in the Leuven NICU, and in part already reported in infants with BPD, EA, and iCDH were pooled and compared with healthy controls (5, 6, 13, 14).

## Methods

This prospective study was approved by the Ethics Committee on clinical studies of the University Hospitals Leuven (Belgium). Informed consent was obtained from all participants' parents prior to the HRIM procedure.

### Cohort Characteristics

For the purpose of analysis, study patients were grouped into four categories:

(1) *Control patients*: Healthy preterm and term infants were recruited as control patients. Pressure-impedance data from term control infants, combined with preterm infants acquired at near-term equivalent age, were used as control data. The healthy, preterm infants were studied as part of a research study investigating maturational trends over time in preterm infants. The manometric studies at term age in preterm infants and the data collected in the healthy term infants were pooled and used as control data for the current study (5, 14). (2) *Preterm infants with BPD*: Infants with grade I, II, and III BPD (15, 16) were eligible for and included in the study if they were still tube fed at term age. Heated, humidified, high nasal flow cannula and oxygen supplementation was not a contra-indication for inclusion, on the condition that the infant was receiving oral feeding as part of their daily clinical care. (3) *iCDH*: Infants born with isolated CDH, i.e., without associated anomalies nor chromosomal abnormalities, were included. (4) *EA*: Infants born with EA and with repair of continuity of their native esophagus were eligible. Patients with chromosomal anomalies or syndromic features were excluded. These last studies were performed at least 6 weeks postsurgical repair. All HRIM studies were performed in the NICU, except for those for EA patients, who were studied at the motility clinic of the University Hospitals Leuven. Use of patch in CDH repair, type of EA (17), age, and weight were recorded.

### High-Resolution Impedance Manometry Recordings

Infants were monitored using oxygen saturation and ECG during all procedures performed in the NICU. A quiet environment was created, lights were dimmed, and parents were asked to comfort their child during the procedure. HRIM recordings were acquired using an 8 Fr (external diameter 2.7 mm) solid-state catheter, incorporating 13 pressure sensors spaced 1 cm apart, and 6 adjoining impedance segments, each 2 cm apart (Unisensor AG, Attikon, Switzerland). This 8 Fr catheter contained a central lumen, which could be used for tube feeding. Pressure and impedance data were acquired at 20 Hz [Solar GI, Laborie, Medical Measurement Systems (MMS), Enschede, The Netherlands]. Oral sucrose 24% was used for procedural analgesia, just before transnasal insertion of the manometric probe. The position of the catheter across the EGJ was verified in real-time, guided by pressure color plots on the screen. During the study, infants were fed expressed breastmilk or formula milk, according to their current diet, in a semi-reclined or lateral position. Normal saline (1/10 dilution of NaCl 0.9%) was added to feed to enhance bolus conductivity for impedance measurement (5, 6, 13). Infants were allowed to drink from a bottle *ad libitum*. Whenever oral bottle feeding proved to be difficult, boluses of 0.5 ml were given orally, using a syringe. All the studies were performed by the same 2 researchers (MR and NR).

### High-Resolution Impedance Manometry Analysis

**Figure 1:**
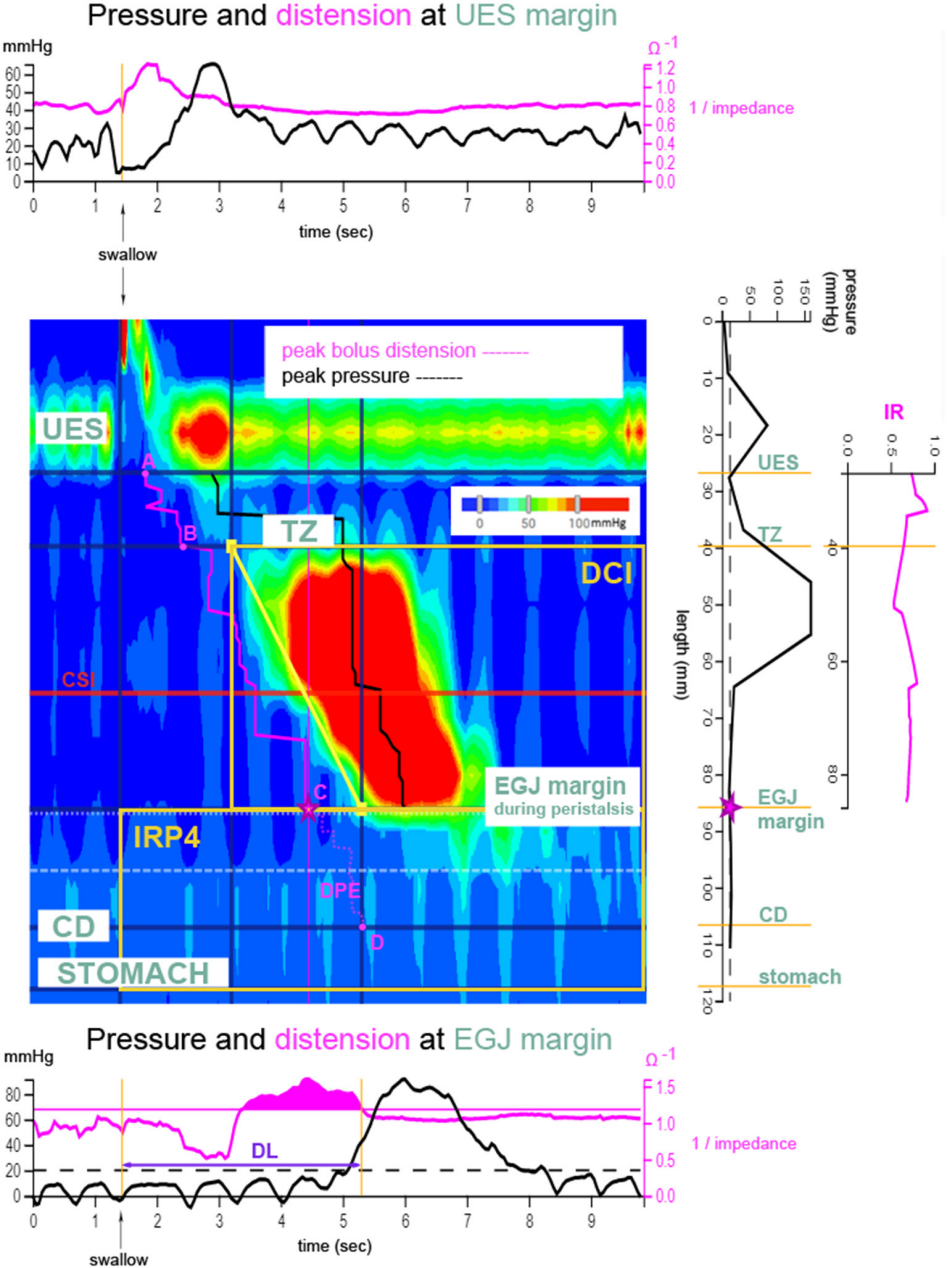
Derivation of esophageal bolus transport metrics. Clouse plot (pressure topography plot, as described in Swallow Gateway™) after a bolus swallow. Pressure amplitudes along the esophagus and at the level of the esophago-gastric junction (EGJ, C) are depicted as a color plot as a function of time. The trajectory of the bolus can be followed using the impedance signal (pink), thereby determining distension of the esophagus. Transition zone (TZ, B) is the zone between striated and smooth muscle. The plots above and below the Clouse plot show the pressure (black) and impedance (pink) recordings at the upper esophageal sphincter (UES, A) and EGJ margins, respectively. The plot to the right of the Clouse plot shows axial pressures recorded along the esophageal body at the exact time point of maximum distension of the esophageal lumen, just proximal to the EGJ (⋆contractile deceleration point). The following esophageal pressure topography metrics are marked on the Clouse plot: distal contractile latency time (DL), distal contractile integral (DCI), and EGJ-integrated relaxation pressure (IRP4). Pressure-impedance derived metrics are shown in pink. Distension pressure during esophageal emptying (DPE) determines the pressure during esophageal emptying from EGJ margin to crural diaphragm (CD, D) (from C to D). Impedance ratio (IR) is depicted on the far right plot. IR is a parameter reflecting bolus clearance and is calculated as the average of all impedance ratio values along the esophageal body from UES to EGJ. Contractile segment impedance (CSI) is the impedance value at peak pressure of the contraction and is measured in the third of the esophagus above the EGJ. Used with permission from (6).

Normal peristaltic wave patterns were defined as the presence of propagating swallows with amplitude >30 mmHg post-deglutition along the esophagus, from the upper esophageal sphincter (UES) to the EGJ margin (18). Analysis of HRIM recordings was performed using the web application, *Swallow Gateway*™ (available at: https://swallowgateway.com/), as previously described (19). HRIM study data were exported from MMS as an ASCII file and subsequently uploaded to the website. Nutritive swallows were selected, guided by the impedance signal at the distal UES margin. The following three EPT metrics were calculated (Figure 1): (1) *distal contractile integral* (DCI), an index of contractile vigor, calculated as the product of the amplitude, duration, and span of the distal esophageal contraction (20); (2) *distal latency* (DL), the interval between UES relaxation and the contractile deceleration point (CDP); (3) *IRP4*, the median integrated relaxation pressure, measured after UES relaxation over the EGJ during 4 seconds of maximal relaxation.

Three categories of esophageal pressure-impedance metrics were derived (Figure 1): (1) Intra-bolus *distension pressure* during *esophageal emptying* (DPE); (2) *impedance ratio* (IR), expresses bolus clearance [luminal clearance is defined by 50% recovery of impedance, relative to baseline (21, 22)]. A higher IR value indicates less effective bolus clearance; and (3) *Contractile segment impedance* (CSI), the impedance value at peak esophageal contraction, measured at the distal one-third above the EGJ margin. CSI reflects esophageal mucosal integrity and is inversely related to esophageal acid exposure (23, 24). For evaluation of EGJ barrier function, mean resting pressure and EGJ-CI were determined at the onset of the manometric recording over three periods, including three respiratory cycles, above a threshold of gastric pressure. The EGJ-contractile integral (EGJ-CI) defines the vigor of the EGJ barrier, independent of time (25). EGJ resting pressure is the pressure measured over the EGJ at rest, not related to swallowing, at the beginning of the study.

### Statistical Analysis

Swallow parameters from each individual patient were calculated as medians. Parameters were predominantly non-parametric, therefore non-parametric statistical methods were used, including the Mann-Whitney U and independent samples Kruskal-Wallis (KW) tests. A two-sided *post-hoc* pairwise between group analysis was performed when the *p*-value of the KW test was < 0.05. Significance values adjusted by Bonferroni correction are reported. Statistical data analysis was conducted using IBM SPSS Statistics (IBM Corp, released 2017, version 25.0, IBM Corp, Armonk, NY). A *p*-value < 0.05 indicated statistical significance.

## Results

Patients were categorized in four groups: 28 infants with BPD, 12 with iCDH, and 10 with EA and their results compared with data from 13 healthy infants. Manometric studies were well-tolerated and no life threatening events were recorded during the procedures. The clinical characteristics of the included patients are presented in Table 1. Comparisons referred to in the table were made across the four patient groups using the KW test.

**Table 1:** Patients characteristics of control patients and NICU patients.

	**Controls term**	**Controls healthy preterm**	**BPD**	**iCDH**	**EA**
Number of patients	3	10	28	12	10
GA (weeks)	37.0 [36.3–38.4]	30.4 [28.4–31.4]	26.2 [24.3–28.3]	38.0 [31.6–39.0]	39.2 [31.9–41.4]
Birth weight (grams)	3,290 [3,220–3,320]	1,300 [670–1,570]	755 [500–1,060]	3,000 [1,140–3,890]	2,735 [1,700–3,615]
PNA at study (days)	62 [31–62]	47 [41–65]	118 [54–171]	27 [14–91]	62 [42–122]
PMA at study (weeks)	45.1 [42.9–45.1]	36.9 [36.0–39.6]	40.6 [37.4–44.1]	43.2 [36.1–51.0]	47.0 [43.3–58.9]
Weight at study (grams)	4,620 [3,800–4,900]	2,536 [2,342–2,890]	2,910 [2,150–4,102]	3,318 [2,450–5,720]	4,250 [2,240–6,180]
Oxygen (*n*)	0	0	10	2	0
HHHFNC (*n*)	0	0	4	1	0

*Median values [range] are displayed. BPD, bronchopulmonary dysplasia; EA, esophageal atresia; GA, gestational age; HHHFNC, heated and humidified high flow nasal cannula; iCDH, isolated congenital diaphragmatic hernia; PMA, postmenstrual age (GA + PNA); PNA, postnatal age*.

Of 12 patients with iCDH, 11 suffered from left-sided hernia. A patch was used to close the gap in 9/12 patients (13). Nine patients with EA had a type C EA (with distal tracheo-esophageal fistula), while one patient had a type A EA. Data from six manometric recordings were excluded from the analysis because of small oral intake, combined with straining (*n* = 5), or because of consecutive swallowing with deglutitive inhibition of peristalsis (*n* = 1). One patient with type A EA was also excluded from statistical analysis because of normal contractility (outlier within the EA group). None of the patients underwent fundoplication prior to the manometric recording.

### Esophageal Motility Patterns

All infants displayed normal esophageal motor patterns, with the exception of infants with EA, in which three different types of motility pattern were observed: complete aperistalsis (3/10 patients), segmental distal contraction (6/10 patients), and normal contractility in one patient with type A EA (1/10 patients).

### EPT and Pressure-Impedance Analysis

EPT parameters and pressure-impedance during bolus swallowing are summarized in Table 2. A total of 224 nutritive swallows were analyzed: 48 in controls and 96, 60, and 20 swallows in patients with BPD, iCDH, and EA, respectively.

**Table 2:** Esophageal pressure topography and pressure-impedance parameters in controls and NICU patient groups.

**Parameter**	**Controls**	**BPD**	**iCDH**	**EA**
**Esophageal pressure topography**
DCI mmHg. cm. s)	316 [132–818]	547 [148–1,420]	357 [203–807]	60 [11–129]^*^
DL (s)	4.64 [3.66–5.89]	4.89 [3.68–8.93]	5.18 [4.06–7.00]	5.20 [4.06–6.34]
IRP4 (mmHg)	10.2 [2.5–22.1]	6.9 [0.4–37.5]	11.8 [0.7–36.0]	9.2 [1.1–14.6]
EGJ resting pressure (mmHg)	36.9 [8.7–70.4]	34.1 [16.5–124.4]	35.5 [17.4–59.4]	22.9 [5.9–70.2]
EGJ-CI (mmHg.cm)	33.4 [6.3–64.7]	34.3 [15.2–136.8]	37.6 [14.6–83.2]	32.0 [4.6–75.2]
**Pressure-impedance analysis**
DPE (mmHg)	12.8 [8.8–24.6]	14.2 [8.9–34.1]	15.5 [8.2–25.3]	20.0 [9.2–25.3]
IR	0.70 [0.56–0.84]	0.67 [0.56–0.84]	0.76 [0.63–0.89]	0.88 [0.55–0.89]
CSI (Ω)	874 [703–1,196]	1,012 [596–1,514]	825 [573–1,069]	1,261 [895–1,627]

*Median values [range] are given*.

* *Independent Samples Kruskal-Wallis test; post-hoc significance vs. BPD and vs. iCDH. BPD, bronchopulmonary dysplasia; CSI, contractile segment impedance; DCI, distal contractile integral; DL, distal latency; DPE, distension pressure esophageal emptying; EA, esophageal atresia; EGJ, esophagogastric junction; EGJ-CI, EGJ-contractile integral; iCDH, isolated congenital diaphragmatic hernia; IR, impedance ratio; IRP4, 4-second integrated relaxation pressure*.

**Figure 2:**
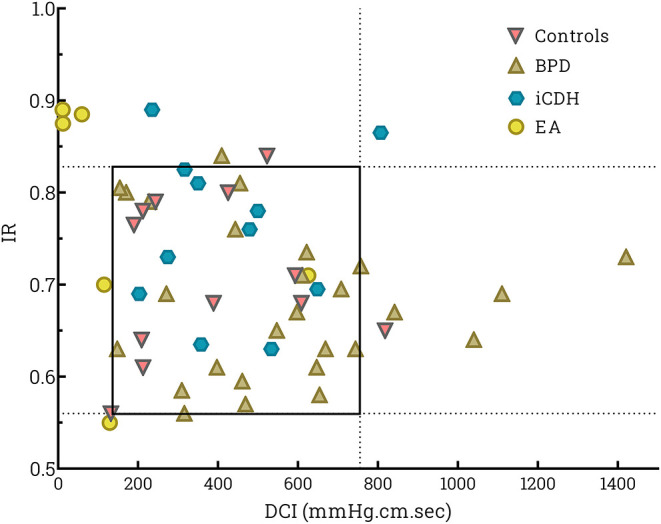
Scatter plot of swallows per individual patient of distal contractile integral (DCI) vs. impedance ratio (IR). DCI determines the strength of the peristaltic contraction, while the IR value reflects bolus clearance. A higher IR indicates less effective bolus clearance in the esophagus. The square indicates the P5–P95 for healthy controls.

Esophageal contractile vigor, determined by DCI, differed significantly in patients with EA compared with other patient groups (KW *p* = 0.001, *post-hoc* analysis BPD *p* < 0.0001 and CDH *p* = 0.02 resp). DCI was significantly lower compared with healthy controls (MWU *p* < 0.0001). The Impedance Ratio (IR) (parameter for bolus clearance), was not statistically different in patients with EA (KW *p* = 0.83). DCI vs. IR was plotted and the P5–P95 area defined in healthy controls (Figure 2). All patients, except 1 infant with EA, had decreased distal esophageal contractile strength, as indicated by lower DCI. Three infants with EA had an associated decrease in bolus clearance capacity (indicated by higher IR). All other patient groups fell within the tentatively “normal” area, except for four infants with BPD who displayed raised DCI and two patients with iCDH and higher IR. Three out of these four patients with BPD had BPD grade III. Patients with EA exhibited the highest CSI values; however, the difference was not significant.

The EGJ was easily recognized on HRIM studies in all patients. No differences were detected among patient groups in EGJ resting pressure, IRP4, or EGJ-CI.

## Discussion

In this study, we aimed to characterize esophageal motility during feeding in different NICU patient groups, using state-of-the-art HRIM and objective esophageal bolus transport metrics. Based on a pooling effort, we explored four patient groups: a control group of healthy infants, infants with BPD, iCDH, and EA.

In the reference group of healthy preterm infants evaluated at near-term age or term neonates, peristaltic esophageal wave sequences were easily recognized in response to nutritive swallows. Peristaltic waves are essential to optimal propagation of a bolus through the esophagus and to clear the esophagus after passage of a bolus. In earlier studies investigating esophageal motility in preterm infants during dry swallows, peristaltic contractions were only registered in ~27% of swallows (26), and this proportion did not increase with increasing postmenstrual age. In more recent studies using water-perfused high resolution manometry, a higher proportion of swallow-induced peristaltic contractions were identified (4, 27). The current study differs from previous published investigations in three major aspects. First, we used a solid-state catheter, thereby improving tolerance and reducing artifacts during procedures. Second, the objective of the current study was to describe esophageal motility during “physiological,” oral feeding, as opposed to dry, spontaneous, or induced swallows. Third, we used state-of-the-art esophageal parameters, thereby increasing the objectivity of the observations.

The first group of patients were preterm infants with BPD. These patients are particularly prone to feeding problems and some remain dependent on tube feeding, even after discharge from hospital (28–30). Knowledge on the underlying reasons for these feeding problems is sparse but are assumed to be multifactorial. Oral aversion, disturbed suck-swallow-breathing coordination, respiratory insufficiency, and gastro-esophageal reflux disease are believed to be contributors to oral intake problems in patient with BPD (3, 29, 31, 32). In the current study, infants with BPD measured at term age showed no differences in esophageal bolus transport mechanisms compared with controls. DCI and IR, markers for esophageal contractile vigor and efficient clearance capacity, respectively, were comparable with those found in controls. Additionally, EGJ-CI values did not differ from those of controls, suggesting that the anti-reflux barrier is well-established in most infants with BPD and that these infants are not at increased risk for pathological gastro-esophageal reflux.

The second group of patients under investigation were born with iCDH. Only one previous study, using low resolution manometry, has investigated esophageal motility in children and young adults with iCDH, and found that almost all patients with CDH appeared to suffer from esophageal dysmotility after repair for CDH (33). The results of the current study are not consistent with the previously published data, as our findings demonstrate similar motility to that characterized in controls. These findings imply normal development of the enteric nervous system, which is essential to generate esophageal contractility. Previously, we have described weaker EGJ end-expiratory barrier pressures in the same cohort of patients with CDH without patch repair and in relation to defect size (13). That study also detected decreased EGJ contractile activity during respiration in patients with CDH who had undergone patch repair. In the present study, the total group of patients with iCDH showed esophageal bolus transport metrics comparable with those of controls. Within this limited dataset, most infants with iCDH appeared to have normal EGJ relaxation after deglutition and efficient anti-reflux barrier function at rest.

The final group of patients studied were those born with EA. Dysphagia or swallowing disorder is very common (15–52%) in patients with EA (34–36). A variety of pathological motor patterns have been identified using HRM in children with EA (37–39); however, the most challenging aspect in diagnosing patients with esophageal dysphagia is the fact that quantitative methods fail to link functional symptoms, like dysphagia, with the observed esophageal motor disorders. Within the current limited dataset, failed peristalsis was observed in 3 of 10 cases and segmental distal contraction in 6 patients, while peristalsis was normal in 1 patient. We noted two major differences in our dataset compared with a previous report from Lemoine et al. (37). First, we did not observe patients with pressurization within our limited dataset. Second, within our dataset, the only patient with type A EA displayed normal contractility, rather than aperistalsis and pressurization, as found in the previous study. As expected, contractile vigor was significantly lower in patients with EA. Further, although IR appeared to be higher (suggesting decreased esophageal clearance capacity), the difference among the four patient groups was not significant. Larger studies are needed to fully explore the clearance capacity of the esophagus above and below the anastomosis. The evaluation of the clearing capacity of the distal esophagus is particularly useful to guide decisions on fundoplication interventions. The distension pressures in the esophagus during accommodation and compartmentalized transport were not increased, suggesting a lack of obstruction at the site of anastomosis.

The present study has some important limitations. First, the collection of reference data from healthy infants; we combined data from 10 healthy preterm infants at term age with those of 3 healthy term controls and we consider these to be the best reference data currently available. Second, the dataset is based on a relatively small number of patients and swallows within each patient group. Only nutritive swallows were selected, based on clear bolus passage. Third, the position of feeding was not standardized. To improve the infant's comfort we chose to feed them in their habitual position (supine or lateral position). Despite these drawbacks, we believe that this study is very valuable. Esophageal motility studies using pressure-impedance analysis are feasible in young infants. This is the first investigation to characterize esophageal motility and EGJ function during nutritive feeding in a neonatal population. Hence, we have been able to describe esophageal pressure-impedance metrics in a control group of healthy preterm and term infants, and compare them with neonates with congenital malformations. It is also important to acknowledge that, although recruitment and motility studies in young infants in a NICU setting is challenging, we successfully achieved the aims of this study.

In future studies we would like to investigate the following hypotheses.

*In infants with BPD:* To study the role of heated and humidified high flow nasal cannula on the esophageal motility parameters and EGJ function in infants with BPD.

*In infants with iCDH:* To further explore the effect of patch vs. no patch on EGJ function.

*In infants with esophageal atresia:* To investigate the differences between proximal and distal esophageal (baseline) impedance. To explore the clearing capacity of the esophagus distal to the anastomosis to guide decisions regarding fundoplication.

*In all neonates*: To investigate the role of body positioning during feeding. To investigate the differences between proximal and distal esophageal (baseline) impedance.

We conclude that esophageal motility studies using pressure-impedance analysis are feasible in young infants. Esophageal motility following nutritive swallows, with adequate bolus transport, appear to be well-established in all investigated NICU patient groups, with the exception of infants with EA, most of whom exhibited ineffective esophageal motility. Deglutitive and non-deglutitive EGJ relaxation was functional in all investigated NICU patient groups.

## Data Availability Statement

The raw data supporting the conclusions of this article will be made available by the authors, without undue reservation.

## Ethics Statement

The studies involving human participants were reviewed and approved by EC University Hospitals Leuven. Written informed consent to participate in this study was provided by the participants' legal guardian/next of kin.

## Author Contributions

MR was the principal investigator for the study. She conceptualized and designed the study. She recruited the study patients, obtained the informed consent from the parents, and included the study patients. She acquired all the data, conducted the data analysis, and performed the statistical analysis. She wrote the first draft of the manuscript and revised the manuscript based on revisions of the co-authors. NR developed the study design, acquired all the data, and oversaw the data analysis. Together with MR, she had access to all the study data, and takes responsibility for the integrity of the data and accuracy of data analysis. She reviewed and supervised the finalization of the manuscript. TO was critical in further developing the study design and providing technical support on the software analysis. He revised the manuscript critically for important intellectual content. KA was critical in further developing the study design and revised the manuscript critically for important intellectual content. VC revised the manuscript critically for important intellectual content. All authors contributed to the article and approved the submitted version.

## Funding

MR was supported by the fund for clinical research, University Hospitals Leuven.

## Conflict of Interest

NR and TO hold a patent on AIMplot, the software used to analyze the pressure flow data. Swallow Gateway™ resource is provided and hosted by Flinders University. The remaining authors declare that the research was conducted in the absence of any commercial or financial relationships that could be construed as a potential conflict of interest.

## Publisher's Note

All claims expressed in this article are solely those of the authors and do not necessarily represent those of their affiliated organizations, or those of the publisher, the editors and the reviewers. Any product that may be evaluated in this article, or claim that may be made by its manufacturer, is not guaranteed or endorsed by the publisher.
